# Editorial: Introduction to the *Toxins* Special Edition Honoring Dr. John D. Groopman for His Contributions to the Field of Aflatoxin Carcinogenesis Research

**DOI:** 10.3390/toxins18020097

**Published:** 2026-02-14

**Authors:** David L. Eaton

**Affiliations:** Department of Environmental Occupational Health Sciences, School of Public Health, University of Washington, Seattle, WA 98195, USA; deaton@uw.edu

It is with both excitement and sorrow that I now write this brief introduction and overview to this Special Edition of Toxins. Excitement in honoring the tremendous, life-long contributions that my friend and colleague, Professor John D. Groopman, has made to our understanding of the important global public health impacts of the common dietary contaminant, aflatoxin B_1_ (AFB_1_)—one of the most potent human carcinogens ever identified. Sorrow, in that my co-editor and long-time friend and colleague, Dr. Thomas W. Kensler, is no longer with us to share the release of this momentous special edition of *Toxins* (please see the memorial article in this Special Issue titled: *Thomas W. Kensler (1948–2025): A Legend in Antioxidant Response Pathways and Aflatoxin Carcinogenesis Research)*.

When I was first asked if I would serve as editor of this Special Edition in honor of Dr. Groopman, I was both honored and enthusiastic to do so, but with one condition: that Dr. Kensler must also be invited as a co-editor so that he can share in the honor and effort needed to bring this to fruition. Tom enthusiastically agreed, and our journey began. Dr. Kensler was a career-long collaborator and friend of Dr. Groopman and contributed greatly to the field of aflatoxin research in his own right. Thus, this Special Issue also honors the lifelong contributions of Dr. Kensler to aflatoxin carcinogenesis research. The papers in this Special Issue have been published over the course of nearly two years of effort on the part of the multiple invited authors. The eight specific articles are divided into two main categories of aflatoxin research: the implications of aflatoxin contamination on public health and the evaluation of current and future approaches to mitigate the effects of aflatoxin contamination globally.

Part A: Public Health Significance of Aflatoxins: Recent Advances in the Toxicology and Epidemiology of Aflatoxins:

This volume begins with an introduction to the field of aflatoxin research, featuring a brief overview of the incredible contributions Dr. Groopman has made. Both Drs. Groopman and Kensler (and many other notable aflatoxin researchers) received their doctoral training in the laboratory of Dr. Gerald Wogan at MIT in the 1970s. Dr. Wogan basically ‘founded’ the field of aflatoxin toxicology, and his legacy lives on through the voluminous, productive research of Drs. Groopman, Kensler, and numerous other Wogan trainees, as well as their many successful students and post-docs, some of whom are co-authors on several of the papers in this Special Issue of Toxins. To put the field—and this Special Issue—in perspective, an introductory article, titled “65 Years on—Aflatoxin Biomarkers Blossoming: Whither Next”, was written by the two co-editors (T.W. Kensler and D.L. Eaton) [1]. This article provides a brief overview of the remarkable scientific gains that have been made over the ~65 years since the initial reports of the fungal mycotoxins dubbed ‘aflatoxins’ (the name is derived from the fungal source (*Aspergillus flavus* toxins) and their fluorescing color under UV light (AFB1—blue, AFG1—green)), with an emphasis on the contributions of Dr. Groopman to our understanding of the global public health implications of dietary contaminations with AFB_1_.

(1) Species differences in biotransformation of AFB_1_.

There are widespread species differences in the hepatotoxic and carcinogenic effects of AFB1. The molecular basis for the toxicity differences between these species of AFB1 is discussed in detail in an article in this Special Issue [2]. Such differences are determined by genetic differences in hepatic cytochrome P450 (CYP)-mediated activation of AFB_1_ to the AFB_1_-8,9-*exo*-eoxide (AFBO) and especially genetic differences in the relative rate of detoxification of AFBO via hepatic glutathione S-transferase enzymes. For example, mice express a form of glutathione S-transferase (mGstA3-3) that has remarkably high affinity for AFBO and is expressed constitutively in the liver, making mice largely resistant to AFB_1_-induced hepatocarcinogenesis, whereas human alpha class GSTs have no measurable detoxifying ability [3,4,5,6,7]. Rats have an orthologous alpha class form to mice with relatively high detoxifying activity toward AFBO, but it is not constitutively expressed in the liver, and thus rats are quite sensitive to the hepatotoxic and carcinogenic effects of AFB_1_. Avians have a wide range of relative sensitivity to AFB1. Domestic turkeys are quite sensitive to the hepatic effects of AFB. Indeed, the initial discovery of aflatoxins was from an outbreak of liver disease in a turkey facility and dubbed ‘Turkey X Disease’ [1,8,9]. Hepatic CYP enzymes effectively activate AFB to AFBO, but an alpha-class GST with significant AFBO activity is not constitutively expressed in the liver of turkeys, similar to rats [10]. Fish, especially salmonids, are remarkably sensitive to the carcinogenic effects of AFB_1_. A single 1 h exposure of rainbow trout embryos to 0.5 ppm AFB_1_ produced liver tumors in 60% of exposed embryos 1 year later [11]. One reason for the extraordinary susceptibility of trout to AFB_1_ is the absence of any GST with significant detoxifying activity toward AFBO and reduced DNA repair capacity [12].

(2) Epidemiology of Aflatoxin carcinogenesis in populations across the globe

The risk of AFB_1_-induced hepatocellular carcinoma has been recognized for decades, especially in populations with endemic hepatitis B virus. Drs. Groopman, Kensler, and co-workers were among the first to establish highly sensitive and specific effects-related biomarkers of aflatoxin exposure, such as AFB-albumin adducts in the plasma and AFB-N7guanine adducts in urine [13]. Many of the early population-based epidemiologic studies of AFB_1_ carcinogenesis occurred in Qidong Province [14]. It was within this highly exposed population that the potent interaction between HBV and AFB_1_ was first demonstrated and where AFB_1_-specific molecular biomarkers were developed by Groopman and Kensler [15,16,17]. Although public health interventions and altered agricultural practices have greatly reduced the AFB_1_-associated cancer burden in China [18], AFB remains a significant public health concern in many other parts of the world. In this Special Issue, Dr. Katherine McGlynn and colleagues at the National Cancer Institute, along with Dr. Groopman, provide an overview of the global estimated exposures to dietary AFB using biomarker data [19]. Of substantial importance is the relatively recent identification of substantial exposures from maize (corn) products in Mexico and Central America, described in detail in this article.

(3) Unique and distinct mutational spectrum of aflatoxin B1 and sterigmatocystin

It has been known since the early days of aflatoxin research that the carcinogenic and hepatotoxic effects of AFB1 are the result of DNA damage and subsequent mutagenesis. Sterigmatocystin (ST), a structural analog of AFB_1_ and a naturally occurring mycotoxin produced by certain strains of *Aspergillus*, is significantly less extensively studied than AFB1. Although both molecules share the difuran moiety with a double bond, where metabolic activation occurs, they differ in the cyclopentanone ring structure area of AFB1 (ST contains a phenolic ring structure in place of cyclopentanone). In this Special Issue, the mutational spectra of metabolically activated AFB_1_ and ST are compared using ultra-high-resolution DNA sequencing techniques [20]. Although the two molecules are both genotoxic, the mutational spectra of the two were quite different. AFB1 mutations were largely focused on G-->T transversions that escaped DNA repair, whereas ST generated fewer G-->T transversions. However, the mutational spectrum generated by activated ST was reflective of more extensive oxidative damage to DNA compared to AFB1. The authors speculate that the detailed mutational spectra for each compound might provide “*unique genomic fingerprints that could be future biomarkers aiding the fields of risk assessment and cancer prevention*”.

(4) Challenges with assessing the potential impact of dietary aflatoxin exposure on childhood development in under-resourced areas of the world

Although the primary public health focus on dietary aflatoxin exposures has focused on liver cancer and, in rare instances, liver toxicity, there are numerous suggestive studies of other adverse effects of aflatoxins, particularly in early life growth and development [21,22,23,24]. It is possible that subtle AFB_1_-induced toxic effects on the liver, especially in malnourished children, could enhance the consequences of malnourishment, leading to decreased growth (stunting). In this Special Issue, this topic is reviewed in detail [24]. Although not a systematic review, the authors evaluated the study design and outcomes of 5 cross-sectional studies, 9 longitudinal studies, and two intervention trials focused on aflatoxin exposure and early childhood growth (stunting). All the studies used AFB_1_-albumin adduct biomarkers to evaluate AFB_1_ exposure. Although many of the studies found associations between AFB_1_-albumin biomarkers and a common measure of stunting (low body height for age), the article discusses the many challenges to comparing such studies. The authors note that many previous reviews often find mixed associations but often lack a critical assessment of study design and little data on patterns of aflatoxin exposure. The paper provides an interesting discussion of the diverse patterns of aflatoxin exposure over time and how such patterns influence study design to address public health consequences of childhood aflatoxin exposure in under-resourced parts of the world.

Part B: Recent Approaches to Mitigation of Dietary Contamination of Aflatoxins:

The identification of aflatoxins as a significant public health concern in many parts of the world has led to the development of strategies to mitigate the potential adverse health impacts of aflatoxin contamination in food staples such as corn (maize) and peanuts. This requires an understanding of both the molecular biology of how aflatoxins are formed by *Aspergillus* spp. and a variety of host-specific factors that determine the extent of mold growth, both pre- and post-harvest.

Three articles in this Special Issue review the status of a wide variety of efforts to mitigate aflatoxin contamination.

(1) Review of approaches to reducing AFB_1_ production, growth of *Aspergillus* on food crops, and modifying host resistance to aflatoxin contamination

In their comprehensive review, Guo et al. [25] examine the historical approaches that have been used to alter the growth of *Aspergillus* and/or aflatoxin production, genetic approaches to modifying host (crop) resistance to mold infection, and recent advances in genome editing for enhancing drought tolerance and increasing plant immune responses. The authors note in their conclusions, “*For decades, the research community has sought the proverbial “Silver Bullet”—a single intervention that could reliably and consistently eliminate preharvest aflatoxin contamination. No individual solution is likely to succeed in isolation. Instead, durable progress will require coordinated, multi-component strategies that combine knowledge of host resistance and breeding, fungal biology, and environmental influences with modern tools such as CRISPR genome editing, biotechnology, nanotechnology, and improve forecasting and detection systems.*” [25].

(2) Approaches to decrease the bioavailability of aflatoxins in food and feed via administration of binding agents

In order to mitigate the risks to public health from dietary aflatoxin exposure, the Phillips laboratory and other investigators have been working for years to identify ways to reduce or eliminate aflatoxin absorption by binding to various clays, which, if effective, could be used *as* AFB_1_ binders in feed and food. This could be beneficial for both public health prevention in areas where food contamination is unavoidable and for reducing the impacts of contaminated feed on livestock. In this Special Issue, the Phillips laboratory (Oladele et al. [26]) discusses recent results of studies on chlorophyll- and chlorophyllin-amended montmorillonite clays for the binding of AFB_1_. Their data demonstrated that the green engineered clays significantly detoxified AFB_1_ (86% to 100%) and provided complete protection at levels as low as 0.1%.

(3) New approaches to aflatoxin mitigation using artificial intelligence

Gamlath and Wu [27] look into the future and discuss how artificial intelligence, combined with the plethora of new genomics tools and biotechnologies, could be used to identify novel approaches to reducing the public health impacts of aflatoxin contamination. They include a discussion of how such new approaches need to be accommodated by regulatory frameworks throughout the world. Using a ‘case study’ of aflatoxin-contaminated corn, they propose a conceptual scenario to demonstrate how AI and biotechnology could be incorporated into the entire food supply chain to manage aflatoxin contamination.
